# The effect of heavy metal contamination on humans and animals in the vicinity of a zinc smelting facility

**DOI:** 10.1371/journal.pone.0207423

**Published:** 2019-10-28

**Authors:** Xiaoyun Shen, Yongkuan Chi, Kangning Xiong

**Affiliations:** 1 School of Life Science and Engineering, Southwest University of Science and Technology, Mianyang, China; 2 State Engineering Technology Institute for Karst Desertification Control, Guizhou Normal University, Guiyang, China; 3 World Bank Poverty Alleviation Project Office in Guizhou, Southwest China, Guiyang, China; Alexandria University, EGYPT

## Abstract

A diagnosis of heavy metal poisoning in sheep living on pastures in the vicinity of a smelting facility in the Wumeng mountain area of China was based on laboratory tests and clinical symptoms. Furthermore, heavy metal contamination in the food chain was found to have a deleterious effect on the health of local residents. The levels of copper(Cu), zinc(Zn), cadmium (Cd), and lead (Pb) in irrigation water, soil, forages, and animal tissues were measured in samples taken from the vicinity of a smelting facility and control samples. Heavy metal contents in food (corn, rice, and wheat), as well as in human tissues (blood and hair) obtained from local residents were also determined. Hematological values were also determined in human and animal samples. The content of Cu, Zn, Cd, and Pb in irrigation water, soils, and forages were markedly higher in affected areas than in samples from healthy pastures. Concentrations of Cd and Pb were 177.82 and 16.61 times greater in forages than controls, respectively, and 68.71 and 15.66 times greater in soils than controls, respectively. The heavy metal content in food (corn, rice, and wheat) from affected areas was markedly higher than in the control samples. Cd and Pb content in the tissues of affected sheep were markedly higher than in control animals (*P < 0*.*01*), while concentrations of Cd and Pb in blood and hair samples from local residents were markedly higher than in control samples (*P < 0*.*01*). The occurrence of anemia in affected humans and animals followed a hypochromic and microcytic pattern. The intake of Cd and Pb was estimated according to herbage ingestion rates. It was found that the levels of Cd and Pb which accumulated in sheep through the ingestion of vegetation growing in the sites closest to the smelter were approximately 3.36 and 38.47 mg/kg body wt./day, respectively. Such levels surpassed the fatal dosages for sheep of 1.13 mg Cd/kg body wt/day and 4.42 mg Pb/kg body wt./day. The serum total antioxidant capacity in affected humans and animals was significantly lower than in the controls (*P < 0*.*01*). Serum protein parameters in affected humans and animals were significantly reduced (*P* < 0.01); therefore, it was concluded that heavy metal contamination caused harm to sheep, and also posed a significant risk to humans living in the vicinity of the zinc smelting facility.

## Introduction

The Wumeng mountain area is located in the Yunnan-Guizhou Plateau in the southwest China, where the three provinces of Guizhou, Yunnan, and Sichuan intersect. The area is an important pasture land for sheep, and sheep farming is vital to the production system in the Wumeng mountain area as animals provide meat, wool, and hides for local residents [1–2]. During the past 10 years, concentrations of lead (Pb), cadmium (Cd), copper(Cu), and zinc(Zn) in the air, water, soil, forages, and food (corn, rice, and wheat) have been increasing in the region. In terms of the potential adverse effects on human and animal health, Pb, Cd, and arsenic have caused the most concern [3–4] because they are readily transferred though food-chains and are not known to serve any essential biological function [5]. Industrial emissions of Cd are the largest source of environmentally hazardous amounts of Cd [6–7], and the most polluting industries are those associated with mining and smelting, followed by manufacturing, with losses of heavy metals from manufactured products during use and when discarded. The reclamation and use of waste products contaminated with Cd can also Pb to pollution [8–9]. Pb is considered to be a major environmental contaminant and has been more widely reported as a cause of accidental poisoning in humans and livestock than any other substance [10]. Multiple manuscripts have reviewed specific aspects of Pb toxicology in humans [10–12] and livestock [7, 13–14]. As an environmental contaminant, Pb is often combined with Cd. Both metals generate similar health effects, and therefore the effects are additive [15–16].

The Wumeng mountain area is also an important production base in the Southwest China for non-ferrous metals. The area has extensive heavy metal reserves, characterized by large quantities of ores containing Zn, Cu, and Pb [2]. A large number of industrial enterprises were established for the purpose of Pb, Zn, Cu, and polymetallic extraction in the 2010s. After a few years of intensive development, metallurgical industries occupied a wide area of former pastures and farmland [1]. A number of sheep grazing on pastures in the vicinity died after the smelters went into operation and all of the affected sheep were characterized by anemia, emaciation, anorexia, depression, and weakness. However, the body temperature, respiratory rate, and heart rate of the affected animals were normal. In the most severely affected area, 48.36% of sheep were affected and the mortality rate reached 70.67%. Local residents also suffered seriously from heavy metal contamination, which has caused major economic losses and has become a serious scourge. Heavy metals have intoxicated local residents through the food chain, interfering with the normal functions of the body and generating serious adverse health effects. However, little research has been undertaken to assess the movement of heavy metal contaminants in the environment, their effects on animal health, and particularly their effects on local residents following the passage of heavy metals through the food chain.

The aim of this study was to determine the relationship between sheep death and the possible environmental impact of the local metallurgical industry. This study also aimed to investigate the effects of heavy metal contamination on human health in individuals living in the vicinity of a zinc smelting facility in the Wumeng mountain area in the Southwest China.

## Materials and methods

### Ethics statement

Sheep were cared for as outlined in the Guide for the Care and Use of Animals in Agricultural Research and Teaching Consortium. Sample collections from animals were approved by the Institute of Zoology, Chinese Academy of Sciences, Institutional Animal Care and Use Committee (Project A0066).

Human subjects research was approved by the State Engineering Technology Institute for Karst Desertification Control Human Subjects Protection Committee, and all participants provided written informed consent.

### Study area

The area studied was located in the Yunnan-Guizhou Plateau of China (25°49´–28°35´N, 102°45´–105°17´E), with an average elevation of 2200 m above sea level. The annual precipitation is 960 mm and the average atmospheric temperature is 10–12°C. The polluted area was located in Hezhang County on the Chinese Yunnan-Guizhou Plateau (26°36´–27°26´N, 103°36´–104°45´E). The control area was located in Dushan County on the Yunnan-Guizhou Plateau (26°29´–27°28´N, 103°33´–104°45´E). The grassland vegetation is mainly Puccinellia (*Chinam poensis ohuji*), Siberian Nitraria (*Nitraria sibirica Pall*), floriated astragalus (*Astragalus floridus*), poly-branched astragals (*Astragalus polycladus*), falcate whin (*Oxytropis falcate*), ewenki autonomous banner (*Elymus nutans*), common leymus (*Leymus secalinus*), and Junegrass (*Koeleria cristata*). Most of the plants are herbaceous and are good resources for grazing animals. The grain crops grown in the area are mainly maize, wheat, and rice.

### Selected humans and animals

Fifteen affected sheep aged 2–3 years were selected from polluted pastures in Xinguanzhai Village of Magu Town in Heizhang County in Guizhou Province in the Chinese Wumeng mountain area. All 15 sheep displayed obvious signs of poisoning, including anemia, emaciation, anorexia, and weakness. Fifteen healthy sheep aged 2–3 years were also selected from healthy pastures in the Guizhou Grassland Technology Extension Station of Dushan County in Guizhou province in China. A clinical examination revealed that all control animals were in good health.

Forty human volunteers aged 20–30 years were selected to participate in the study. Twenty lived in a polluted area of Xinguanzhai Village in Magu Town in Heizhang County, Guizhou Province, in the Chinese Wumeng Mountain Area. All of these volunteers displayed obvious signs of anemia. The other 20 healthy volunteers lived in an uncontaminated area of the Guizhou Grassland Technology Extension Station in Dushan County, Guizhou Province, in the Chinese Yunnan-Guizhou Plateau.

### Sample collection

Animal blood samples (15 ml) were obtained from the jugular vein of all sheep. Human blood samples (15 ml) were obtained from the median cubical vein of ten human volunteers, using 1% sodium heparin as an anticoagulant and stored at −10°C prior to the analysis of heavy metals. Wool was taken from the neck of all sheep. Hair was taken from the head of 20 human volunteers, washed and degreased as described by [17] and kept in a desiccator over silica gel prior to analysis.

For all sheep, general anesthesia was used, including intramuscular injection of 2% serazole hydrochloride (1 ml/kg body wt), 6 minutes interval, followed by an intramuscular injection of 4% sodium phenobarbital (1 ml/kg body wt). Six minutes after anesthesia was induced, all sheep were killed by exsanguination and samples of at least 40 g were taken from the lobus caudatus of the liver, renal cortex of the right kidney, left ventricle of the heart, spleen, lobes of the lung, a gluteal muscle of the left posterior limb, last rib, radius of the left forelimb, and molar teeth. Samples were packed in labelled plastic bags and immediately transported to the laboratory. Visible fat, connective tissue, and major blood vessels were removed from the soft tissues, which were then dried at 80°C for 48 h, ground by a mortar, passed through a 0.5 mm sieve, and then stored in a desiccator over silica gel.

**Fig 1:**
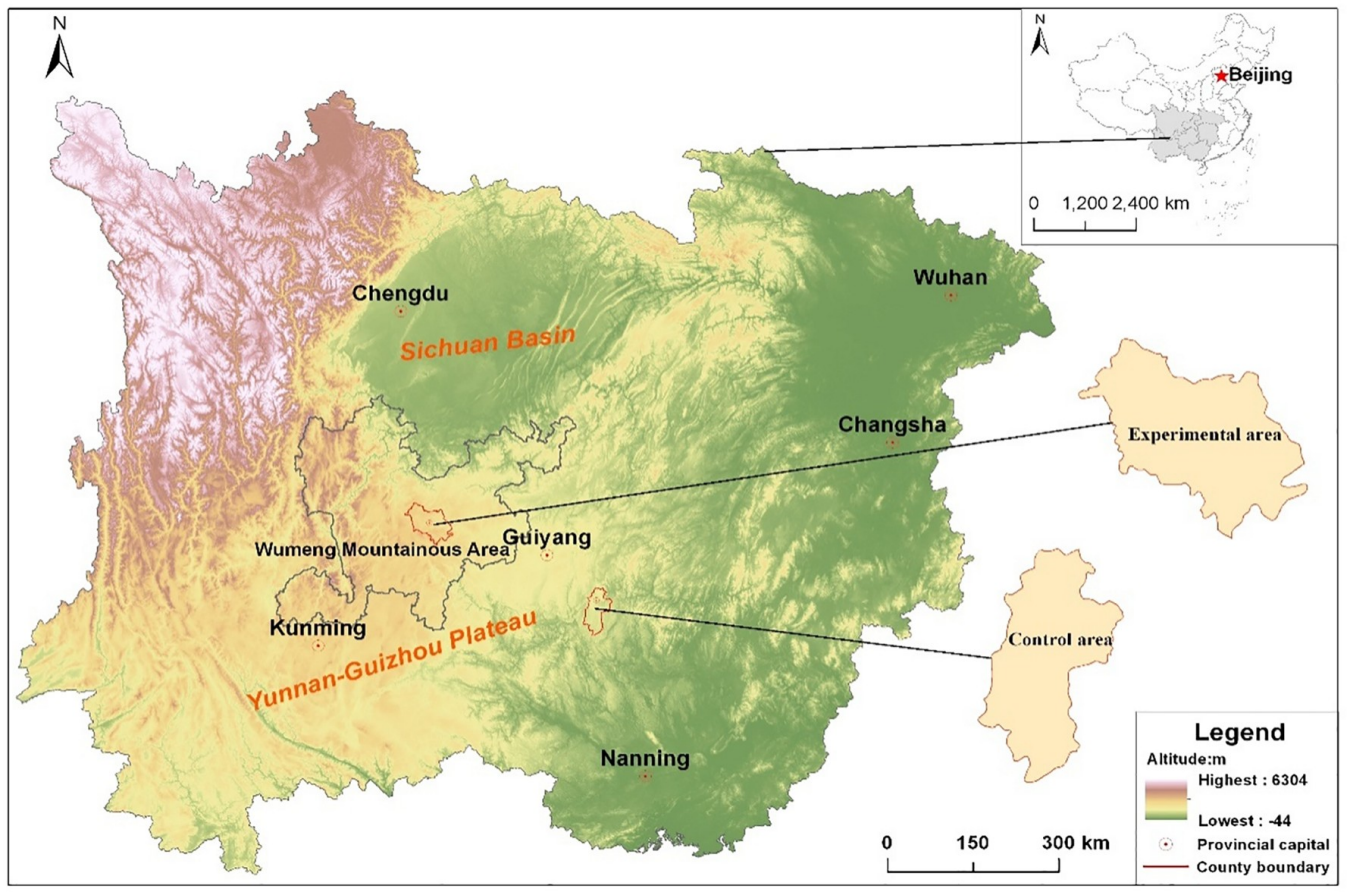
Study area on the Yuannan-Guizhou Plateau.

**Fig 2:**
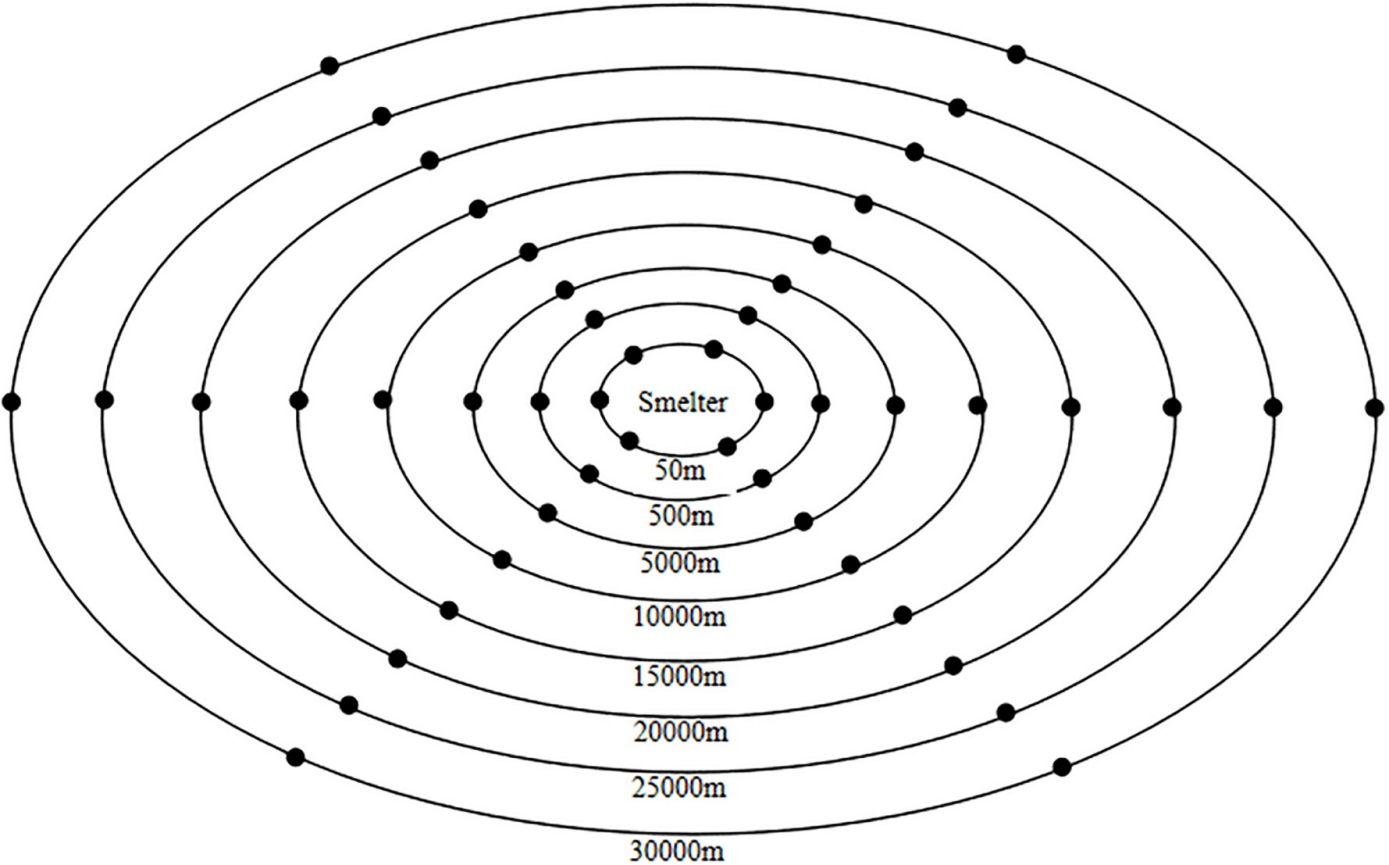
Schematic of the sampling distribution for soil, water, forage, and food samples from the affected pasture. Sampling sites: 50, 500, 5,000, 10,000, 15,000, 20,000, 25,000, and 30,000 m from the smelting facility.

Samples of water, soil, and herbage were taken at 48 sampling sites situated at distances of 50–30,000 m from the zinc smelter (Fig 1). Multiple small portions of herbaceous vegetation were cut from the pasture in this area and mixed. To reduce soil contamination, the forage samples were cut 1 cm above ground level. The herbage samples were dried at 80°C for 48 h and ground by a mortar to facilitate chemical analysis [18–19]. Soil samples were taken from the surface layer (0–30 cm) of the pastures using a 30-mm diameter cylindrical corer. Soil samples were dried at 80°C for 48 h and passed through a 5 mm sieve. Water samples for irrigating the pasture and farmland from the smelters and food samples (grains of rice, corn, and wheat) were also collected from the farmland of a local resident. Food samples were dried at 80°C for 72 h, ground by a mortar, passed through a 0.5 mm sieve and stored in a desiccator over silica gel (Fig 2). Control samples of water, soil, forage, and food were collected from Dushan County in the the Yunnan-Guizhou Plateau, the Southwest China.

### Hematological and biochemical examination

Hemoglobin, packed cell volume, red blood cell, white blood cell, neutrophil, lymphocyte, eosinophil, basophil, and monocyte levels were determined using an automated hematology analyzer (SF-3000, Sysmex-Toa Medical Electronic, Kobe, Japan). Ceruloplasmin, lactate dehydrogenase, aspartate aminotransferase, alanine aminotransferase, alkaline phosphatase, creatinine, cholesterol, blood urea nitrogen, glutathione peroxidase (GSH-Px), superoxide dismutase (SOD), malondialdehyde, total antioxidant capacity, sodium, potassium, magnesium, calcium, and inorganic phosphorus levels in serum were determined using an automated biochemical analyzer (Olympus AU 640, Olympus Optical Co., Tokyo, Japan). Serum protein (total protein, albumin, and globulin) electrophoretic studies were performed on cellulose acetate using the EA-4 electrophoresis apparatus (Shanghai Medical Apparatus and Instruments Factory, Shanghai, China). All biochemical serum values were measured at 25°C.

## Analysis of heavy metals

For each analysis, 1 g of sample (1 ml of blood) was added to 1 ml of hydrogen peroxide and 3 ml of nitric acid. Samples were digested at 180°C in fluoro-plastic vessels in sealed containers to avoid the loss of vapor, and therefore, there was no reduction in the volume of the resulting digest. Molybdenum was determined using flameless atomic absorption spectrophotometry (3030 graphite furnace with a Zeeman background correction; Perkin-Elmer, Waltham, MA, USA) [18–19]. Pb, Cd, Cu, Zn, and manganese(Mn) concentrations were determined using atomic absorption spectrophotometry (AA-640; Shimadzu Co., Ltd, Tokyo, Japan). The accuracy of the analytical values was confirmed by comparing to certified elemental concentrations in reference materials from the International Atomic Energy Agency and National Institute of Standards (Bovine liver SRM 1577a). The limited accuracy of samples with very low concentrations resulted in concentrations below a particular threshold being recorded as ‘trace’, given that zero measurements were difficult to demonstrate.

### Statistical analysis

Data were analyzed using the statistical package for the social sciences (SPSS, version 20.0; SPSS Inc., Chicago, IL, USA), and presented in the form of the mean ± standard error (SE). Significant differences between groups were assessed using Student’s *t*-test with least significant differences of 1% (*P < 0*.*01*).

## Results

All of the affected sheep were characterized by characterized by anemia, emaciation, anorexia, depression, and weakness. In the most severely affected area, 48.36% of sheep were affected and the mortality rate reached 70.67%. Local residents also suffered by heavy metal contamination. The main signs is anemia, emaciation, and weakness in all affected farmers. All cases have been mild, and no deaths occurred. However, the body temperature, respiratory rate, and heart rate of the affected humans were healthy.

**Fig 3:**
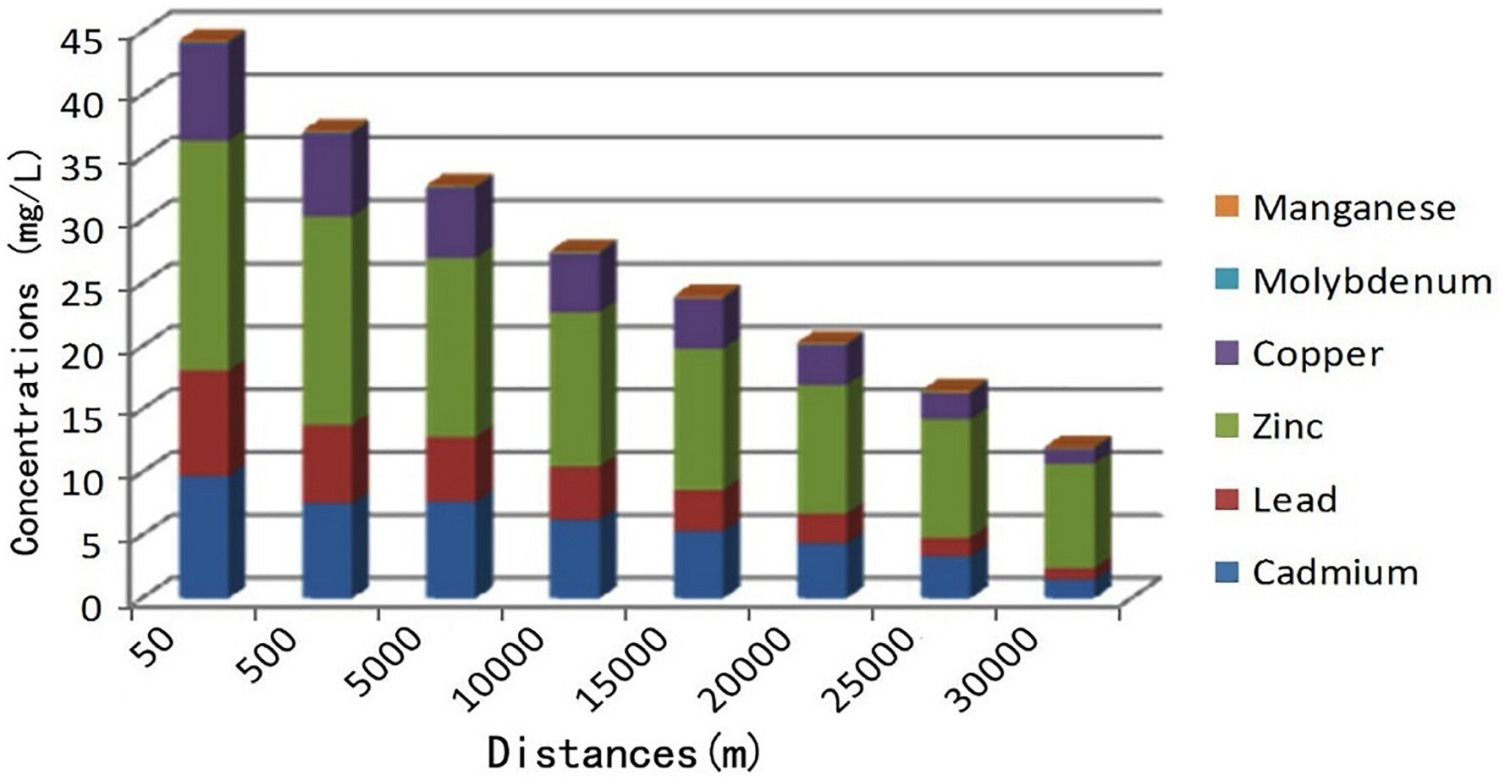
Relationship between heavy metal concentrations in irrigation water (mg/l) and the distance of the sampling sites from the smelting facility.

**Fig 4:**
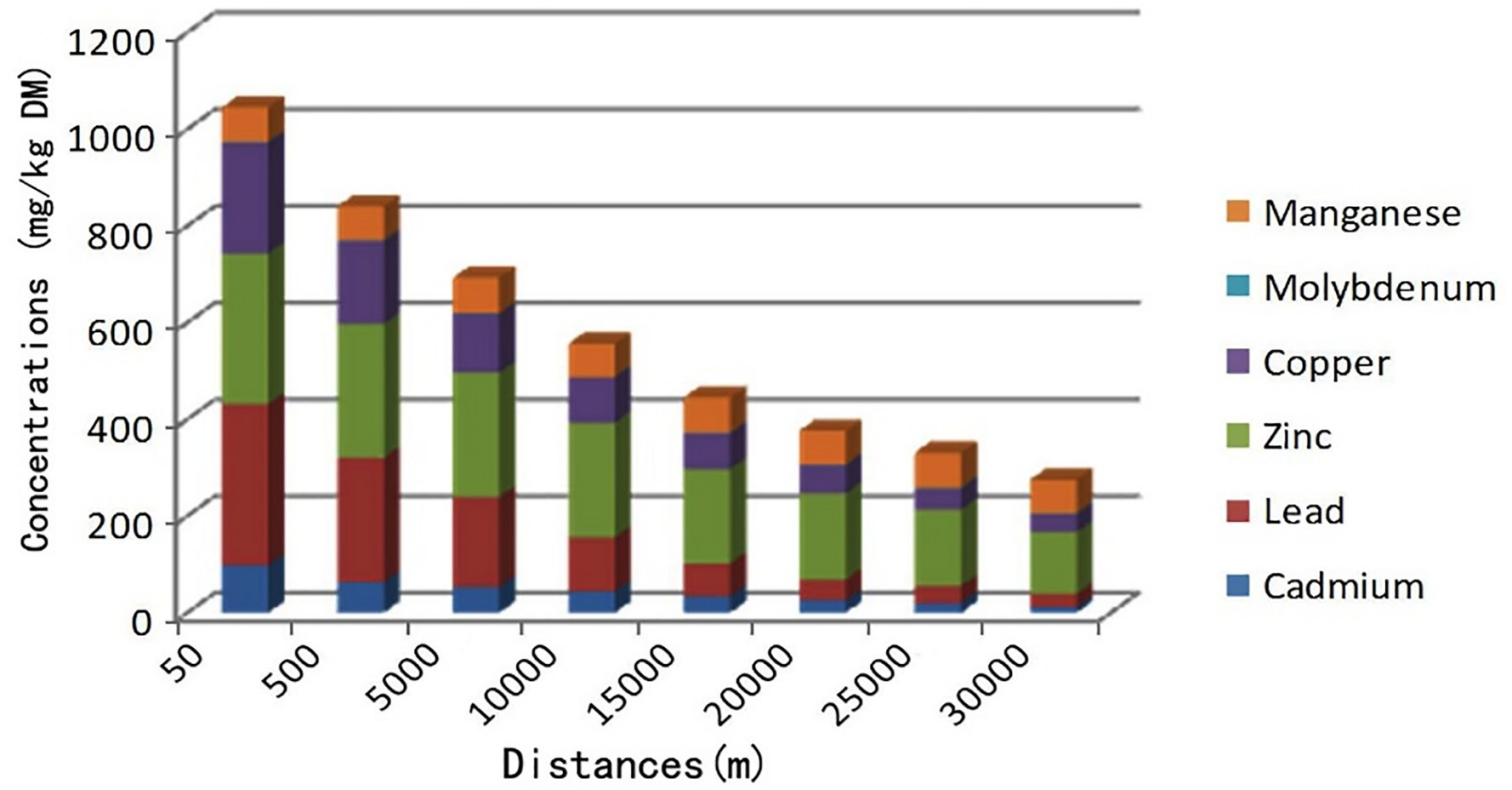
Relationship between heavy metal concentrations in soils (mg/kg DM) and the distance of the sampling sites from the smelting facility.

**Fig 5:**
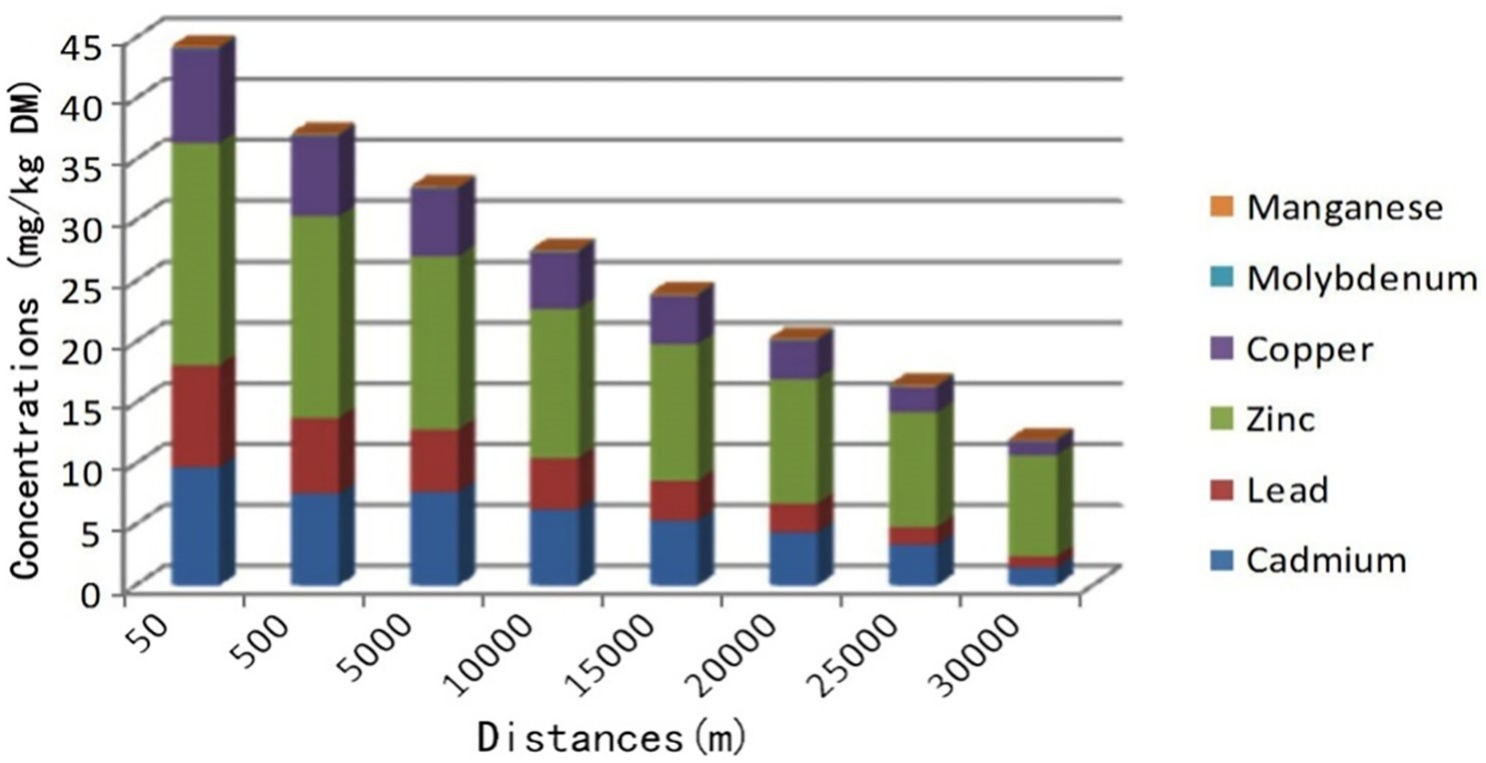
Relationship between heavy metal concentrations in forages (mg/kg DM) and the distance of the sampling sites from the smelting facility.

It was found that heavy metal concentrations clearly decreased with increasing distance from the zinc smelting facility (Figs 3–5). Heavy metal concentrations (Pb, Cd, Cu, and Zn) in irrigation water, soils, forages, and food (corn, rice, and wheat) in affected pastures were markedly higher than those in the control area (*P < 0*.*01*; Tables 1 and 2). The mean Cd and Pb concentrations in affected pastures exceeded the control levels by 68.71 and 15.66 times in soil, respectively, and 556.15 and 16.61 times in herbage, respectively. Considering that sheep were grazing exclusively in affected pastures, Cd and Pb ingestion rates were estimated (Table 3). The estimation was based on an average herbage ingestion of 76.3 g (d.w)/kg body wt./day in the sheep [20]. The ingestion rates were in the range of 3.27–38.47 mg/kg body wt./day and 0.19–3.36 mg/kg body wt./day for Pb and Cd, respectively.

**Table 1:** Metal concentrations in soil, irrigation water, and herbage in all samples (mg/kg).

Metals	Irrigation water		Soil		Forage	
	Affected water	Control	Affected soil	Control	Affected forage	Control
Cd	5.81±0.65^a^	0.004±0.001	43.29±4.67^a^	0.63±0.05	7.23±0.55^a^	0.013±0.003
Pb	3.89±0.35^a^	0.047±0.006	132.35±8.17^a^	8.45±0.77	104.78±8.12^a^	6.31±0.95
Zn	12.57±1.93^a^	0.015±0.003	218.16±9.27^a^	32.37±1.91	178.01±6.36^a^	23.48±2.67
Cu	4.33±0.63^a^	0.017±0.005	103.32±5.98^a^	11.37±1.21	21.67±2.37^a^	6.37±1.27
Mn	0.12±0.02	0.112±0.013	63.85±6.37	61.53±4.39	17.95±3.39	17.89±3.76
Mo	0.11±0.02	0.097±0.011	1.57±0.21	1.66±0.61	1.22±0.13	1.25±0.27

Cd = cadmium, Pb = lead, Zn = zinc, Cu = copper, Mn = manganese, Mo = molybdenum

^a^ *P <* 0.01

**Table 2:** Metal concentrations in corn, rice, and wheat in all samples (mg/kg).

Metals	Corn		Rice		Wheat	
	Affected corn	Control	Affected rice	Control	Affected wheat	Control
Cd	0.113±0.013^a^	0.013±0.003	0.124±0.011^a^	0.014±0.002	0.152±0.011^a^	0.012±0.003
Pb	0.74±0.07^a^	0.14±0.03	1.53±0.02^a^	0.17±0.02	1.73±0.11^a^	0.19±0.03
Zn	27.14±2.53^a^	17.14±1.53	33.53±1.32^a^	13.57±1.32	35.46±2.43^a^	15.46±1.41
Cu	22.97±1.19^a^	2.97±0.19	12.35±0.14^a^	2.37±0.14	23.15±2.34^a^	3.35±0.34
Mn	4.55±0.31	4.35±0.34	3.73±0.31	3.85±0.31	4.31±0.28	4.12±0.28
Mo	0.57±0.03	0.58±0.04	0.96±0.12	0.97±0.12	0.93±0.12	0.95±0.11

Cd = cadmium, Pb = lead, Zn = zinc, Cu = copper, Mn = manganese, Mo = molybdenum

^a^ *P <* 0.01

**Table 3:** The ingestion rates and fatal dosages in animals and humans (mg/kg body wt./day).

Metals	The lowest ingestion rate		The highest ingestion rate		Minimum cumulative fatal dosage	
	Animal	Human	Animal	Human	Animal	Human
Cd	0.19	ND	3.36	ND	1.13	ND
Pb	3.27	ND	38.47	ND	4.42	ND

Cd = cadmium, Pb = lead, ND = no available data

Content of Pb, Cd, Zn, and Cu in wool, blood, heart, lung, liver, muscle, spleen, and bone of affected sheep were markedly higher than those in healthy animals (*P < 0*.*01*; Tables 4 and 5). Pb and Cd content mainly accumulated in the kidney, liver, and skeleton of affected sheep. Pb, Cd, Zn, and Cu concentrations in blood and hair samples from affected local residents were significantly higher than those in the controls (*P < 0*.*01*; Table 6).

**Table 4:** Metal concentrations in wool and blood samples from sheep (mg/kg).

Metals	Wool		Blood	
	Affected animal	Control	Affected animal	Control
Cd	2.282±0.131^a^	0.361±0.032	0.391±0.022^a^	0.013±0.007
Pb	3.761±0.213^a^	1.161±0.121	0.411±0.021^a^	0.042±0.007
Zn	119.97±7.23^a^	87.61±5.97	14.82±0.97^a^	9.81±2.16
Cu	9.87±0.23^a^	3.73±0.57	1.74±0.07^a^	0.79±0.12
Mn	4.89±0.47	4.63±0.36	0.38±0.03	0.35±0.02
Mo	2.17±0.12	2.18±0.13	0.19±0.07	0.15±0.05

Cd = cadmium, Pb = lead, Zn = zinc, Cu = copper, Mn = manganese, Mo = molybdenum

^a^ *P <* 0.01

**Table 5:** Metal concentrations in heart, lung, liver, kidney, muscle, spleen, rib, radius, and tooth samples from sheep.

Metals	Heart		Lung		Liver	
	Affected animal	Control	Affected animal	Control	Affected animal	Control
Cd	0.39±0.03^a^	0.11±0.02	3.12±0.25^a^	0.67±0.05	7.62±0.56^a^	0.47±0.02
Pb	4.76±0.67^a^	1.34±0.23	4.45±0.38^a^	1.55±0.27	16.37±1.57^a^	0.73±0.05
Zn	137.63±6.56^a^	103.92±7.23	143.87±±8.67^a^	107.82±7.63	233.93±8.72^a^	137.79±8.57
Cu	29.81±3.43^a^	14.72±0.93	25.23 ±2.32^a^	11.67±1.69	258.17±7.93^a^	117.67±7.73
Mn	3.67±0.33	3.52±0.23	3.82±0.27	3.83±0.31	4.73±0.72	4.56±0.59
Mo	1.32±0.11	1.33±0.17	1.53±0.16	1.68±0.37	1.43±0.13	1.39±0.18
Metals	Kidney		Muscle		Spleen	
	Affected animal	Control	Affected animal	Control	Affected animal	Control
Cd	27.37±1.53^a^	0.73±0.29	0.82±0.03^a^	0.18±0.04	0.57±0.05^a^	0.33±0.03
Pb	38.57±2.87^a^	0.77±0.13	2.85±0.52^a^	0.87±0.03	3.87±0.53^a^	0.93±0.11
Zn	157.16±7.43^a^	113.75±7.69	141.13±7.62^a^	113.56±9.87	137.93±9.17^a^	118.62±9.86
Cu	27.21±3.27^a^	11.32±1.73	8.97±0.77^a^	5.83±0.96	9.52±0.57^a^	4.47±0.56
Mn	4.68±0.57	4.73±0.85	3.83±0.57	3.82±0.67	2.56±0.37	2.61±0.29
Mo	1.87±0.22	1.79±0.12	1.28 ±0.18	1.36±0.28	0.79±0.14	0.37±0.03
Metals	Rib		Radius		Tooth	
	Affected animal	Control	Affected animal	Control	Affected animal	Control
Cd	4.77±0.23^a^	0.53±0.07	5.62±0.73^a^	0.78±0.04	5.57±0.54^a^	0.43±0.03
Pb	14.53±2.73^a^	2.37±0.03	22.53±2.51^a^	2.57±0.73	27.82±2.73^a^	1.21±0.02
Zn	97.83±3.43^a^	77.75±3.69	117.13±5.62^a^	83.56±9.87	137.37±7.59^a^	113.97±7.61
Cu	8.21±0.57^a^	4.32±0.73	11.17±0.77^a^	4.83±0.96	8.53±0.87^a^	5.73±0.67
Mn	2.17±0.32	2.09±0.32	1.37 ±0.48	1.36±0.11	1.76±0.31	1.69±0.27
Mo	3.68±0.35	3.73±0.35	4.23±0.53	3.82±0.27	4.22±0.53	4.17±0.76

Cd = cadmium, Pb = lead, Zn = zinc, Cu = copper, Mn = manganese, Mo = molybdenum

^a^ *P <* 0.01

**Table 6:** Metal concentrations in human hair and blood samples (mg/kg).

Metals	Hair		Blood	
	Affected human	Control	Affected human	Control
Cd	1.88±0.12^a^	0.17±0.02	0.29±0.01^a^	0.012±0.001
Pb	2.71±0.33^a^	0.53±0.31	0.31±0.03^a^	0.032±0.003
Zn	99.63±7.71^a^	67.63±6.76	14.82±0.81^a^	6.81±0.76
Cu	8.82±0.67^a^	3.21±0.53	1.34±0.07^a^	0.69±0.01
Mn	4.17±0.37	4.27±0.32	0.37±0.05	0.35±0.04
Mo	2.23±0.15	2.19±0.11	0.17±0.03	0.15±0.02

Cd = cadmium, Pb = lead, Zn = zinc, Cu = copper, Mn = manganese, Mo = molybdenum

^a^ *P <* 0.01

Hematological parameters in affected local residents and sheep are given in Table 7. Compared with healthy controls, hemoglobin levels and packed cell volumes were markedly reduced (*P < 0*.*01*). Those abnormal blood indices indicated a hypochromic microcytic anemia in affected humans and animals. Serum biochemical parameters in affected humans and animals are given in Table 8. Compared with healthy humans and animals, creatinine, lactate dehydrogenase, SOD, and GSH-Px activities were significantly reduced (*P < 0*.*01*). Serum total antioxidant capacity levels in affected humans and animals were significantly lower than those in the controls (*P < 0*.*01*). Malondialdehyde levels in serum from affected humans and animals were significantly higher than those in the controls. Serum protein parameters in affected humans and animals are given in Table 9. Compared with healthy humans and animals, the total protein, albumin, α-globulin, β-globulin, and γ-globulin levels were significantly reduced (*P < 0*.*01*).

**Table 7:** Hematological parameters in animals and humans.

Parameters	Animal		Human	
	Affected animal	Control	Affected human	Control
Hb (g/l)	97.83±6.56^a^	124.56±9.65	87.65±6.56^a^	131.78±9.57
RBC (10^12^/l)	12.36±2.65	12.15±3.66	4.31±0.56	4.53±0.43
PCV (%)	31.21±3.23^a^	39.13±3.15	42.57±4.36^a^	45.17±4.56
WBC(10^9^/l)	6.38±1.27	6.56±1.13	5.18±0.77	5.36±0.63
Neutrophils (%)	56.98±5.87	57.56±5.18	63.98±6.81	65.46±5.38
Lymphocytes (%)	29.79±2.31	30.17±2.19	35.72±3.31	33.27±3.17
Eosinophils (%)	6.42±0.58	6.56±0.67	7.12±0.98	7.46±0.87
Basophils (%)	0.48±0.03	0.51±0.02	0.52±0.07	0.57±0.03
Monocytes (%)	7.13±0.64	6.97±0.67	5.76±0.57	5.36±0.43

Hb = hemoglobin, PCV = packed cell volume, RBC = red blood cells, WBC = white blood cells

^a^ *P < 0*.*01*

**Table 8:** Serum biochemical parameters in animals and humans.

Parameters	Animal		Human	
	Affected animal	Control	Affected human	Control
Cp (mg/l)	51.37±6.67	51.76±5.26	56.36±6.29	55.37±4.17
LDH (U/l)	2.51±0.32^a^	3.67±0.43	2.73±0.37^a^	3.57±0.23
AKP (U/l)	76.78±7.98	77.89±6.56	87.18±7.96	86.78±7.23
AST (U/l)	38.73±3.36	37.93±4.57	38.27±4.22	37.73±3.31
ALT (U/l)	13.36±3.27	13.88±3.77	22.31±3.18	22.36±3.36
BUN (U/l)	6.62±1.37	6.57±1.76	6.57±0.97	6.33±0.61
SOD (U/l)	111.35±8.73^a^	157.67±9.69	123.23±9.77^a^	143.37±9.38
GSH-Px (U/l)	287.67±9.38^a^	389.56±9.77	233.62±7.18^a^	353.72±7.49
CAT (U/l)	16.63±1.25	16.92±1.63	19.68±1.73	19.63±1.52
T-AOC (U/l)	3.01±0.13^a^	4.32±0.17	3.31±0.14^a^	4.37±0.12
CRT (U/l)	257.87±13.27^a^	318.67±7.83	277.51±13.89^a^	336.52±11.35
Chol (U/l)	2.77±0.31	2.81±0.36	2.83±0.37	2.79±0.31
MDA (nmol/l)	11.37±0.38^a^	6.36±0.15	12.53±0.39^a^	7.23±0.57
K (mmol/l)	3.99±0.37	4.03±0.58	3.75±0.38	3.35±0.37
Na (mmol/l)	129.35±5.89	121.76±8.37	116.35±5.33	113.37±4.31
Ca (mmol/l)	2.28±0.21	2.31±0.23	2.52±0.17	2.72±0.19
IP (mmol/l)	1.89±0.32	1.85±0.26	1.63±0.12	1.57±0.17
Mg (mmol/l)	0.89±0.11	0.91±0.12	0.73±0.03	0.81±0.04

Cp = ceruloplasmin, LDH = lactate dehydrogenase, AKP = alkaline phosphatase, AST = aspartate aminotransferase, ALT = alanine aminotransferase, BUN = blood urea nitrogen, SOD = superoxide dismutase, GSH-Px = glutathione peroxidase, CAT = catalase, MDA = malondialdehyde, T-AOC = total antioxidant capacity, CRT = creatinine, Chol = cholesterol, K = potassium, Na = sodium, Ca = calcium, IP = inorganic phosphorus, Mg = magnesium.

^a^ *P < 0*.*01*

**Table 9:** Serum protein concentrations in animals and humans.

Parameters	Animal		Human	
	Affected animal	Control	Affected human	Control
Total protein (g/l)	55.86±4.23^a^	65.54±5.97	57.73±3.92^a^	63.51±5.97
Albumin (g/l)	43.28±2.36^a^	49.79±3.73	45.15±3.76^a^	47.78±3.14
α-Globulin (g/l)	1.79±0.16^a^	2.77±0.23	1.73±0.22^a^	2.67±0.27
β- Globulin (g/l)	3.23±0.33^a^	4.25±0.57	3.39±0.31^a^	4.46±0.43
γ- Globulin (g/l)	7.56±0.68^a^	8.73±0.72	7.46±0.67^a^	8.62±0.82
A/G	3.44±0.31	3.16±0.23	3.59±0.33	3.03±0.32

A = albumin, G = globulin

^a^ *P < 0*.*01*

## Discussion

The contamination of pastures and farmlands with heavy metals (Cd, Pb, Cu, and Zn) may occur in the vicinity of smelting facilities. The maximum tolerable dietary content of Cd and Pb has been set at 0.5 and 30 mg/kg, respectively, for livestock [13]. Cd and Pb concentrations in soil, herbage, and irrigated water samples in affected pastures significantly exceeded the maximum tolerable dietary levels in this study (*P < 0*.*01*). Pb, Cd, Cu and Zn concentrations in those samples decreased markedly with increasing distance from the smelting facility in the Wumeng mountain area. This finding has also been observed in other studies of heavy metal contamination in areas surrounding smelting industries [21], and indicates the aeolian dispersion of particles containing heavy metals (Pb, Cd, Cu, and Zn) from such smelters are a source of heavy metal contamination in soils [22–23]. Waste water discharged from the smelting facilities has been used to irrigate surrounding pastures and farmlands and was therefore also a source of heavy metal contamination (Pb, Cd, Cu, and Zn) in the agricultural soils of the study area.

As an environmental contaminant, Pb is often associated with Cd, since both elements have similar properties and their health effects are additive [21, 24]. In this study, Pb, Cd, Cu, and Zn concentrations in the tissues (wool, blood, heart, lung, liver, muscle, spleen, and bone) of affected sheep were markedly higher than those in controls (*P < 0*.*01*). Hypochromic microcytic anemia was also evident in affected animals. It is generally believed that high skeletal Pb and Cd concentrations are characteristic of chronic exposures to Pb and Cd [25–27]. The cortex of the kidneys of affected sheep contained higher Pb and Cd concentrations than livers. Cd and Pb also accumulated in the bones of affected sheep. All such data are consistent with previous studies of livestock that indicated that those tissues are the critical organs for Pb and Cd accumulation [28–30]. It was therefore concluded that heavy metal contamination due to industrial activities in the vicinity of a zinc smelting facility in the Wumeng mountain area had resulted in serious harm to sheep health.

As a result of activities conducted in smelting facilities in the Wumeng mountain area, a large increase in Cd, Pb, Cu and Zn concentrations was observed in the surrounding soils and herbage. Considering that the sheep were fed exclusively with forage from this pasture, the ingested heavy metal rates were estimated to be in the range of 3.27–38.47 and 0.19–3.36 mg/kg body wt./day for Pb and Cd, respectively. The registered values for the minimum cumulative fatal dosage for sheep are estimated at 4.42 and 1.13 mg/body wt./day for Pb and Cd, respectively [14, 31]. Therefore, the ingestion of forages growing in this pasture, especially in the sites closest to the smelting facility constituted a clear Cd and Pb toxicity hazard for livestock. Pb and Cd intake levels in the affected pasture surpassed the fatal dosage (*P < 0*.*01*). As a consequence of the Cd and Pb uptake, elemental concentrations in tissues (wool, blood, heart, lung, liver, muscle, spleen, and bone) in affected sheep surpassed the critical and control concentrations. Such findings confirmed the potential toxicity of the pasture [32,33]. Sheep were predominantly fed locally grown fodder or grazed in the pasture in the vicinity of the zinc smelting facility and are the primary livestock species exposed to heavy metal contamination in the area. Therefore, the determination of Cd, Pb, Cu and Zn concentrations in domestic animals in that area is important for assessing the potential effects of pollutants on livestock, and for quantifying contaminant uptake by humans.

Pb, Cd, Cu, and Zn concentrations in blood and hair samples from humans living in the vicinity of the zinc smelting facility were also significantly higher than those in the controls (*P < 0*.*01*). Hypochromic microcytic anemia was also evident in affected humans. The levels of SOD, GSH-Px, and total antioxidant capacity are given in Table 8.

Serum total antioxidant capacity is an integrative index used to reflect the antioxidant capacity of the body [34–36]. Little is known about the effects of Pb and Cd on the total antioxidant capacity of sheep. Our results indicated that the total antioxidant capacity levels of affected humans and animals were significantly reduced (*P < 0*.*01*), and the enhanced peroxidation of lipids in intracellular and extracellular membranes resulted in damage to cells, tissues, and organs. SOD and GSH-Px are important antioxidant enzymes that protect against this process [37]. The SOD catalyzes the destruction of the superoxide radical, with potential toxicity arising from dismutation and hydrogen peroxide formation, while The GSH-Px catalyzes the conversion of hydrogen peroxide to water and directly reduces tissue injury from lipoperoxidation [34, 38]. A significant decrease in the activity of either enzyme would therefore cause an increase in free radicals; thus, injuring the corresponding tissues. The results show that SOD and GSH-Px activities in the serum of affected humans and animals were markedly decreased (*P < 0*.*01*). Thus, it can be seen that heavy metal contamination not only resulted in harm to sheep health, but also entered the human body through the food chain and interfered with the normal functions of the body, resulting in harm to human health in individuals living in the vicinity of the zinc smelting facility.

In this study, concentrations of Cu and Zn in soil, herbage, and food were markedly higher in samples from the affected area than those from the control area (*P < 0*.*01*). In general, the maximum tolerable concentrations in sheep were 25 and 300 mg/kg for Cu and Zn, respectively [13,39–40]. Thus, it appears that the heavy metal poisoning of the sheep in the affected pasture was not related to Cu and Zn.On the other hand, an increased intake of Cd interferes with the absorption, accumulation and utilization of iron(Fe),Manganese (Mn) and selenium(Se) and so on, replacement of Cu and Zn in proteins, and decreasing the bio-activity of protein[41–42]. Se element is located the active center of GSH-Px, and each mole of GSH-Px contains 4 g atoms Se in seleno-cysteine (SeCys) residues[41]. Cu, Zn, Mn and Fe in the body are essential for the activity of numerous enzymes, Based on the metal co-factor used by the enzyme, the SODs are broadly categorised in to three distinct groups viz, Fe-SOD, Mn-SOD and Cu/Zn-SOD, that are distributed in different compartments of the cell[42–43]. The Cu and Zn ions play essential roles in Cu/Zn-SOD[43]. The Fe and Mn ion play also important roles in Fe-SOD and Mn-SOD, respectively[44].

## References

[1] Liao JJ, Shen XY, Huo B, Xiong KN. Effect of nitrogenous fertilizer on the antioxidant systems of Wumeng semi-fine wool sheep in the Karst mountain areas. Acta Prataculturae Sinica.2018; 27(1):169–176.

[2] Shen XY, Chi YK, Huo B, Wu T, Xiong KN. Effect of fertilization on ryegrass quality and mineral metabolism in grazing the Wumeng semi-fine wool sheep. Fresen Environ. Bull. 2018; 26 (10): 6824–6830.

[3] Benson NU, Asuquo FE, Williams AB, Essien JP, Ekong CI, Akpabio O, et al Source evaluation and trace metal contamination in benthic sediments from equatorial ecosystems using multivariate statistical techniques. Plos One. 2016; 11(6), e0156485 10.1371/journal.pone.0156485 27257934 PMC4892471

[4] Willscher S, Jablonski L, Fona Z, Rahmi R, Wittig J. Phytoremediation experiments with helianthus tuberosus under different pH and heavy metal soil concentrations. Hydrometallurgy. 2017; 168(3), 153–158.

[5] Ciazela J, Siepak M, Wojtowicz P. Tracking heavy metal contamination in a complex river-oxbow lake system: Middle Odra Valley, Germany/Poland. Sci. Total. Environ.2018; 616(11), 996–1006. 29103644 10.1016/j.scitotenv.2017.10.219

[6] Casalino E, Calzaretti G, Sblano C, Landriscina C. Molecular inhibitory mechanisms of antioxidant enzymes in rat liver and kidney by cadmium. Toxicology.2002; 179, 37–50. 10.1016/s0300-483x(02)00245-7 12204541

[7] Zhang G, Bai J, Zhao Q, Jia J, Wen X. Heavy metals pollution in soil profiles from seasonal-flooding riparian wetlands in a Chinese delta: Levels, distributions and toxic risks. Phys. Chem. Earth.2017; 97(2): 54–61.

[8] Greaves WW, Rom WN, Lyon JL, Varley G, Wright DD, Chiu G. Relationship between lung cancer and distance of residence from nonferrous smelter stack effluent. Am. J. Ind. Med.2010; 2(1), 15–23. 10.1002/ajim.4700020105 7349031

[9] Gąsiorek M, Kowalska J, Mazurek R, Pająk M. Comprehensive assessment of heavy metal pollution in topsoil of historical urban park on an example of the Planty Park in Krakow (Poland). Chemosphere.2017; 179(11),148–158. 28365500 10.1016/j.chemosphere.2017.03.106

[10] Latif R, Malek M, Mirmonsef H. Cadmium and lead accumulation in three endogeic earthworm species. Bull. Environ. Contam. Toxicol. 2013; 90(4), 456–459. 10.1007/s00128-012-0941-z 23283534

[11] Ramachandra TV, Sudarshan PB, Mahesh MK, Vinay S. Spatial patterns of heavy metal accumulation in sediments and macrophytes of bellandur wetland, bangalore. J. Environ. Manage. 2018; 206(7), 1204–1210. 29157887 10.1016/j.jenvman.2017.10.014

[12] Shin W, Choung S, Han WS, Hwang J, Kang G. Evaluation of multiple prps’ contributions to soil contamination in reclaimed sites around an abandoned smelter. Sci. Total Environ. 2018; 642, 314–321. 10.1016/j.scitotenv.2018.06.031 29906722

[13] USA, National Research Council, Subcommittee on Mineral Toxicity in Animals. Mineral tolerance of domestic animals. National Academy of Sciences. 2018; 193–196.

[14] Shen XY, Zhang RD. Studies on "stiffness of extremities disease" in the yak (bos mutus). J. Wildlife. Dis. 2012; 48(3), 542–547. 10.7589/0090-3558-48.3.542 22740519

[15] Satarug S, Baker JR, Urbenjapol S, Haswellelkins M, Reilly PE, Williams DJ, et al A global perspective on cadmium pollution and toxicity in non-occupationally exposed population. Toxicol. Lett. 2003; 137(1), 65–83. 12505433 10.1016/s0378-4274(02)00381-8

[16] Steindor KA, Franiel IJ, Bierza WM, Pawlak B, Palowski BF. Assessment of heavy metal pollution in surface soils and plant material in the post-industrial city of katowice, poland. Environ. Lett. 2016; 51(5), 371–379. 10.1080/10934529.2015.1120509 26809744

[17] Salmela S, Vuori E, Kilpi ÖJO. The effect of washing procedures on trace element content of human hair. Anal. Chim. Acta. 1981; 125(01), 131–137.

[18] Shen XY, Du GZ, Li H. Studies of a naturally occurring molybdenum-induced copper deficiency in the yak. Vet. J. 2006; 171(2), 352–357. 10.1016/j.tvjl.2004.11.006 16490720

[19] Shen XY. Effect of nitrogenous fertilizer treatment on mineral metabolism in grazing yaks. Agr. Sci. China. 2009; 8(3), 361–368.

[20] Shen XY, Zhang J.H, Zhang RD. Phosphorus metabolic disorder of Guizhou semi-fine wool sheep. Plos One. 2014; 9(2), e89472 10.1371/journal.pone.0089472 24586803 PMC3929721

[21] Xie Y, Chen TB, Lei M, Yang J, Guo QJ, Song B, et al Spatial distribution of soil heavy metal pollution estimated by different interpolation methods: accuracy and uncertainty analysis. Chemosphere. 2011; 82(3), 468–476. 10.1016/j.chemosphere.2010.09.053 20970158

[22] Kapusta P, Łukasz S. Effects of heavy metal pollution from mining and smelting on enchytraeid communities under different land management and soil conditions. Sci. Total Environ. 2015; 536(1), 517–526. 26233783 10.1016/j.scitotenv.2015.07.086

[23] Tang W, Zhang W, Zhao Y, Zhang H, Shan B. Basin-scale comprehensive assessment of cadmium pollution, risk, and toxicity in riverine sediments of the haihe basin in north china. Ecol. Indic. 2017; 81, 295–301.

[24] Tella M, Bravin MN, Thuriã SL, Cazevieille P, Chevassus-Rosset C, Collin B.,et al Increased zinc and copper availability in organic waste amended soil potentially involving distinct release mechanisms. Environ. Poll. 2016; 212, 299–306. 10.1016/j.envpol.2016.01.077 26854699

[25] Berglund M, Akesson A, Bjellerup P, Vahter M. Metal-bone interactions. Toxicol. Lett. 2000; 112(113), 219–225. 10720734 10.1016/s0378-4274(99)00272-6

[26] Norouzi S, Khademi H, Cano AF, Acosta JA. Biomagnetic monitoring of heavy metals contamination in deposited atmospheric dust, a case study from Isfahan, Iran. J. Environ. Manage. 2016; 173(10), 55–64. 26974238 10.1016/j.jenvman.2016.02.035

[27] Olawoyin R, Schweitzer L, Zhang K, Okareh O, Slates K. Index analysis and human health risk model application for evaluating ambient air-heavy metal contamination in chemical valley sarnia. Ecotox. Environ. Safe. 2018; 148, 72–81. 10.1016/j.ecoenv.2017.09.069 29031119

[28] Hong S, Candelone JP, Patterson CC, Boutron CF. History of ancient copper smelting pollution during roman and medieval times recorded in green land ice. Science.1669; 272(5259), 246–249.

[29] Edwards JR, Prozialeck WC. Cadmium, diabetes and chronic kidney disease. Toxicol. Appl. Pharmacol. 2009; 238(3), 289–293. 10.1016/j.taap.2009.03.007 19327375 PMC2709710

[30] Hambach R, Lison D, D’Haese P, Weyler J, François G, Schryver AD, et al Adverse effects of low occupational cadmium exposure on renal and oxidative stress biomarkers in solderers. Occup. Environ. Med. 2013; 70(2), 108–113. 10.1136/oemed-2012-100887 23104735

[31] Huang Y, Chen Q, Deng M, Japenga J, Li T, Yang X, et al Heavy metal pollution and health risk assessment of agricultural soils in a typical peri-urban area in southeast china. J. Environ. Manage. 2018; 207, 159–168. 10.1016/j.jenvman.2017.10.072 29174991

[32] Brun LA, Maillet J, Hinsinger P, Pépin M. Evaluation of copper availability to plants in copper-contaminated vineyard soils. Environ. Pollut. 2001; 111(2), 293–302. 10.1016/s0269-7491(00)00067-1 11202733

[33] Hu H, Rothenberg S, Schwartz BS. The epidemiology of lead toxicity in adults: measuring dose and consideration of other methodologic issues. Environ. Health. Persp. 2007; 115(3), 455–462. 10.1289/ehp.9783 PMC1849918 17431499

[34] Hussain T, Shukla GS, Chandra SV. Effects of cadmium on superoxide dismutase and lipid peroxidation in liver and kidney of growing rats: in vivo and in vitro studies. Pharmacol. Toxico. 1987; 60(5), 355–358. 10.1111/j.1600-0773.1987.tb01526.x 3615346

[35] Elmissiry MA, Shalaby F. Role of β-carotene in ameliorating the cadmium-induced oxidative stress in rat brain and testis. J. Biochem. Mol. Toxic. 2000; 14(5), 238–243. 10.1002/1099-0461(2000)14:5<238::AID-JBT2>3.0.CO;2-X 10969995

[36] Obida CB, Alan GB, Duncan JW, Semple KT. Quantifying the exposure of humans and the environment to oil pollution in the niger delta using advanced geostatistical techniques. Environ. Int. 2018; 111, 32–42. 10.1016/j.envint.2017.11.009 29169077

[37] Regoli F, Principato G. Glutathione, glutathione-dependent and antioxidant enzymes in mussel, mytilus galloprovincialis, exposed to metals under field and laboratory conditions: implications for the use of biochemical biomarkers. Aquat. Toxicol. 1995; 31(2), 143–164.

[38] Sazuka Y, Tanizawa H, Takino Y. Effect of adriamycin on the activities of superoxide dismutase, glutathione peroxidase and catalase in tissues of mice. Jpn. J. Cancer Res. 1989; 80(1), 89–94. 10.1111/j.1349-7006.1989.tb02250.x 2496064 PMC5917681

[39] Khalid S, Shahid M, Niazi NK, Murtaza B, Bibi I, Dumat C. A comparison of technologies for remediation of heavy metal contaminated soils. J. Geochem. Explor. 2017; 247–268.

[40] Jacob JM, Karthik C, Saratale RG, Kumar SS, Prabakar D. Kadirvelu K, Pugazhendhi A. Biological approaches to tackle heavy metal pollution: a survey of literature. J. Environ. Manage. 2018; 217, 56–70. 10.1016/j.jenvman.2018.03.077 29597108

[41] Suttle NF. The mineral nutrition of livestock. 4th ed CABI Publishing Cambridge, USA; 2010 pp. 255–459.

[42] Dehury B, Sarma K, Sarmah R, Sahu J. In silico analyses of superoxide dismutases (SODs) of rice (Oryza sativa L.). J. Plant Biochem. Biotechnol, 2013; 22(1):150–156.

[43] Filiz E, Koc I, Ozyigit II. Comparative Analysis and Modeling of Superoxide Dismutases (SODs) in Brachypodium distachyon L, 2014; 173(5):1183–1196. 10.1007/s12010-014-0922-2 24781980

[44] Gracjana L, Trzebuniak K F, Paulina Z P. The activity of superoxide dismutases (SODs) at the early stages of wheat deetiolation. PLOS ONE, 2018;13(3):e0194678 10.1371/journal.pone.0194678 29558520 PMC5860746

